# Functional Consequences of Necdin Nucleocytoplasmic Localization

**DOI:** 10.1371/journal.pone.0033786

**Published:** 2012-03-19

**Authors:** Anat Lavi-Itzkovitz, Marianna Tcherpakov, Zehava Levy, Shalev Itzkovitz, Francoise Muscatelli, Mike Fainzilber

**Affiliations:** 1 Department of Biological Chemistry, Weizmann Institute of Science, Rehovot, Israel; 2 Institut de Neurobiologie de la Méditerranée, INSERM U901, Parc Scientifique de Luminy BP 13, Marseille, France; The Centre for Research and Technology, Greece

## Abstract

**Background:**

Necdin, a MAGE family protein expressed primarily in the nervous system, has been shown to interact with both nuclear and cytoplasmic proteins, but the mechanism of its nucleocytoplasmic transport are unknown.

**Methodology/Principal Findings:**

We carried out a large-scale interaction screen using necdin as a bait in the yeast RRS system, and found a wide range of potential interactors with different subcellular localizations, including over 60 new candidates for direct binding to necdin. Integration of these interactions into a comprehensive network revealed a number of coherent interaction modules, including a cytoplasmic module connecting to necdin through huntingtin-associated protein 1 (Hap1), dynactin and hip-1 protein interactor (Hippi); a nuclear P53 and Creb-binding-protein (Crebbp) module, connecting through Crebbp and WW domain-containing transcription regulator protein 1 (Wwtr1); and a nucleocytoplasmic transport module, connecting through transportins 1 and 2. We validated the necdin-transportin1 interaction and characterized a sequence motif in necdin that modulates karyopherin interaction. Surprisingly, a D234P necdin mutant showed enhanced binding to both transportin1 and importin β1. Finally, exclusion of necdin from the nucleus triggered extensive cell death.

**Conclusions/Significance:**

These data suggest that necdin has multiple roles within protein complexes in different subcellular compartments, and indicate that it can utilize multiple karyopherin-dependent pathways to modulate its localization.

## Introduction

Necdin is a MAGE family protein mostly expressed in the nervous system, muscle and skin, which acts as a cell cycle regulator and plays a role in the differentiation or survival of both central and peripheral neurons [1], [2]. It is also one of several proteins genetically inactivated in individuals with Prader-Willi syndrome (PWS) [3], [4]. Although necdin loss does not necessarily lead to the full spectrum of PWS symptoms [5], necdin-null mice do phenocopy various aspects of the human disease [6]–[9]. Studies on the functional role(s) of necdin have been hampered by the lack of any known catalytic domains in the protein, directing attention to interacting partners as possible leads to mechanistic understanding [10]. Indeed, a diversity of necdin interactors has been described in the literature, including nuclear [11]–[13], cytoplasmic [14]–[16] and plasma membrane proteins [7], [17]. Thus, it is not clear if necdin's primary role is as a cytoplasmic signaling adaptor or as a nuclear transcription modulator.

In order to obtain a more comprehensive overview of the necdin interactome, we carried out a large-scale protein interaction screen with necdin as bait, identifying over 60 new candidate necdin binders. Integration of these interactions into a comprehensive necdin-centered network showed that it naturally decomposes into modules with distinct functions and subcellular localizations. The analysis revealed both nuclear and cytoplasmic modules and a nucleocytoplasmic transport module containing transportin1 and 2, members of the importin β family of nucleocytoplasmic transport factors. Exclusion of necdin from the nucleus affects cellular viability, and characterization of the necdin-transportin interaction indicate that necdin can utilize multiple karyopherin-dependent pathways to modulate its subcellular localization.

## Results

### 1. A necdin interaction network

**Figure 1:**
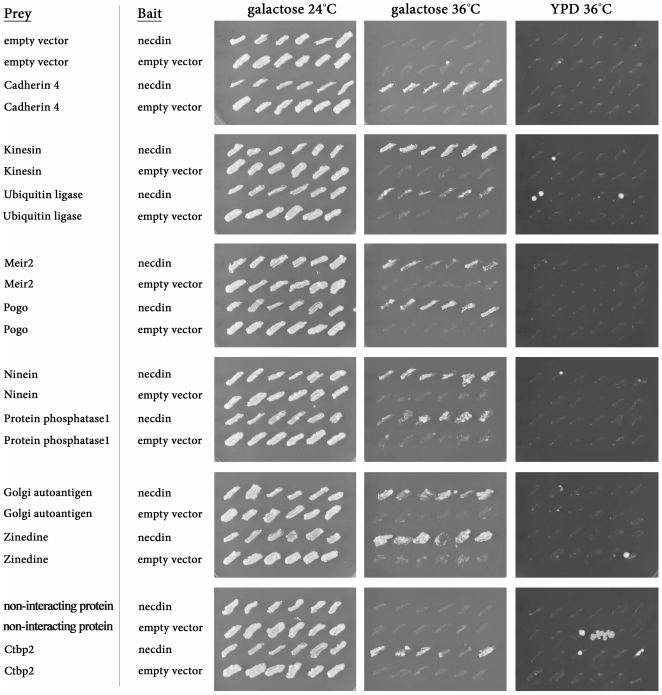
Yeast RRS screen with necdin as bait. Representative pictures of yeast *cdc25-2* colonies after transformation with different candidates at the permissive temperature (24°C), and at the restrictive temperature (36°C). All colonies grow on galactose medium at 24°C, while only colonies expressing candidates that interact with the bait can grow on galactose at 36°C, but not on YPD (prey candidates are under a galactose promoter).

Necdin, like other MAGE family proteins, does not have any known intrinsic catalytic activity and may signal by recruiting other molecules. In order to obtain a comprehensive view of necdin interaction partners, we used the RRS method [18] to screen a mouse embryonic head cDNA library with necdin as bait, and identified 66 candidate interactors (Figure 1, Table S1). Among these 66 candidates, ten have no known function, and two are previously characterized necdin interactors – nucleobindin1 [16] and E1A-like inhibitor of differentiation 1 (Eid1) [11].

**Figure 2:**
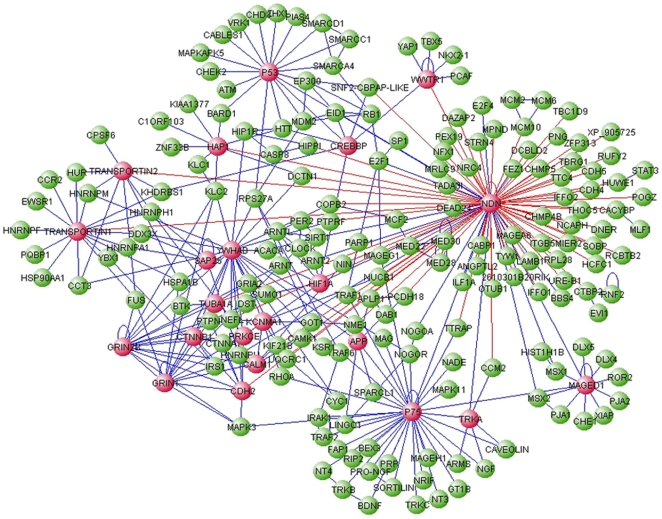
The necdin network comprising first and second degree necdin neighbors. The necdin (Ndn) network was assembled based on the mouse IntAct database, complemented with published necdin interactors and our RRS results. The network contains 205 proteins and 346 interactions. Blue edges denote published interactions, red edges are interactions detected in the present screen. Nodes with more than 5 interactions are marked in red.

We combined our screen data with a comprehensive network of published mammalian protein-protein interactions [19], complemented with a literature search for all previously published necdin interactors. The network contains 2687 proteins and 3817 interactions (Figure S1), and displays a heavy-tailed degree distribution as commonly seen in protein-protein interaction networks [20]. To explore the role of necdin within this network we projected the global network onto the two immediate tiers of interactors - the set of necdin's immediate interactors and the interactors of these immediate interactors. This resulted in a network with 205 proteins and 346 interactions, hereby termed the necdin network (Figure 2, Table S2).

### 2. The necdin network decomposes into functionally coherent modules

**Figure 3:**
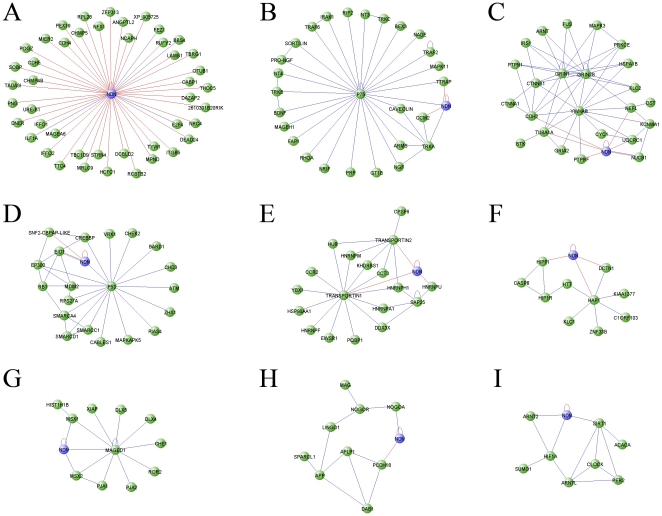
Module decomposition of the necdin network. Modules were detected using the betweenness-centrality clustering algorithm [21]. Only modules containing more than 8 nodes are displayed. Blue edges denote published interactions, red are interactions detected in the present screen. Note that necdin-Eid1 and necdin-Nucleobindin1 are connected with 2 color edges. Modules - **A**. necdin; **B**. p75; **C**. Grin-Ywhab; **D**. p53-Crebbp; **E**. Transportin; **F**. Huntingtin; **G**. MAGE-D1; **H**. APP; **I**. Clock. Necdin (Ndn) is shown for each module, together with interactions with the module members.

An overview of the necdin network reveals a modular structure with binding partners of diverse functional roles and cellular localizations such as the transcription factor p53, the nuclear transcription activator CREBBP, the membrane bound receptor p75 and the cytoplasmic adaptor MAGE D1. We applied a network-partitioning algorithm that divides the network into groups or modules, in a way that optimizes the modularity parameter Q [21]. Partitions with a high Q divide the network into modules with a high intra-group and low inter-group connectivity, sorting into nine structurally coherent modules (Figure 3), most of which are also supported by enriched GO annotations (Table S3). For example, a cytoplasmic module (Figure 3F) includes the Huntingtin protein (HTT) and some of its interactors, such as Hap1 and Hip1r. The present screen uncovered several new links for necdin within this module, including interactions with Dynactin (DCTN1), Hippi and Hap1. A nuclear module (Figure 3D) focused around transcription regulation and chromatin remodeling includes p53 and CREB binding protein (CREBBP) and its regulators Eid1 and Srcap. The interaction between necdin and Eid1 was also reported by Bush et al. [11]. Thus, our screen supports potential roles for necdin in both nuclear and cytoplasmic compartments of the cell, and again raises the question of how such differing roles and interactions can be regulated. A possible solution is provided by a nucleocytoplasmic transport module (Figure 3E), consisting of the karyopherin family members transportins 1 and 2, and a number of their immediate interactors. This module also includes hnRNPU, an RNA binding protein that was previously shown to interact with necdin [22].

### 3. Necdin interacts with Transportins

**Figure 4:**
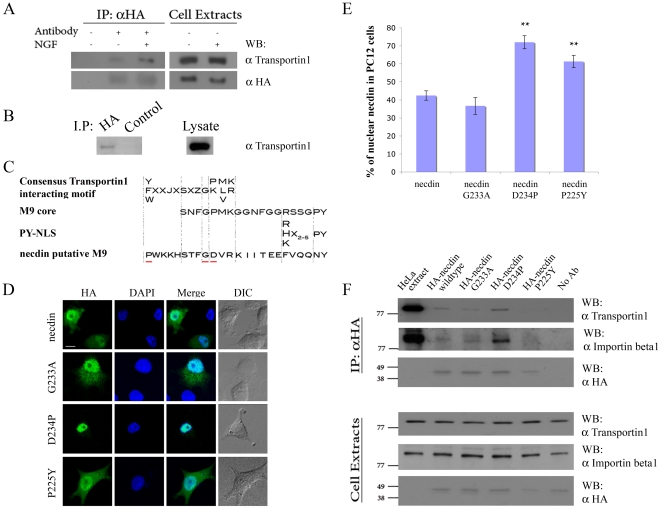
Necdin interacts with Transportin1 via a non-classical M9 motif. **A**. HA-necdin i.p. from transfected PC12 cells, followed by Western blot for transportin-1. Cells were transferred to serum-free DMEM 48 hr after transfection, incubated for 2 hr and then supplemented with or without 150 ng/ml NGF for another 2 hr in serum-free DMEM before lysis. **B**. HA-necdin i.p. compared to control irrelevant antibody i.p. from transfected HEK293 cells, followed by transportin-1 Western blot. 1 mg total cell lysate was used as input for each pull-down in Panels A or B, and 50 µg total lysate was run separately to verify equal input (right lanes). **C.** A putative transportin-interacting sequence in necdin compared to previously published M9 motifs. Residues targeted for mutation are underlined in red. **D**. Representative confocal images of PC12 cells after transfection with HA-necdin mutants (P225Y, G233A, or D234P). Cells were fixed and stained with anti-HA and DAPI 48 hours after transfection. Scale bar 10 µm. **E**. Quantification of nuclear localization of necdin wild type or mutants in PC12 cells, * denotes p<0.05, ** denotes p<0.01, n = 16. **F**. Anti-HA pull-down of HEK293 cell lysates transfected as indicated, followed by Western blot for transportin 1 or importin β1. 1 mg total cell lysate input per pull-down, 50 µg lysate was run separately to verify equal input (lower lanes).

Transportin 2 was found to interact with necdin in our RRS screen. Transportin 1 shares ∼84% sequence homology with transportin 2 in most species [23], and in siRNA experiments we observed compensatory upregulation of the non-targeted transportin isoform in PC12 cells, suggesting functional redundancy (data not shown). We therefore examined necdin-transportin 1 interaction in transfected PC12 (Figure 4A) or HEK (Figure 4B) cells, and observed co-precipitation that is enhanced upon incubation with NGF.

Although necdin has no known NLS or transportin-interaction motifs, we identified a sequence stretch in necdin amino acids 225–248 with some similarity to the proposed consensus for a transportin-interacting M9 motif [24]–[26] (Figure 4C). In order to examine whether this region indeed mediates the necdin interaction with transportin1, we generated a number of mutants, including a glycine to alanine mutation at position 233 (G233A), which is predicted to disrupt the consensus completely and therefore eliminate the interaction [24], [26]. Other mutants were generated to bring the necdin sequence closer to the consensus, including D234P and P225Y. We transfected PC12 cells with each of these mutants and examined necdin localization (Figure 4D), quantifying the ratio of necdin in the nucleus versus the cytosol (Figure 4E). The G233A mutation did not significantly change necdin nuclear localization, indicating that the interaction does not fit characteristics of the known M9 consensus. However, both the P225Y and D234P mutations significantly increased necdin nuclear localization, supporting the functionality of these amino-acids in modulating the interaction with necdin.

To further study the effect of these residues on the interaction of necdin with other members of the karyopherin family, we transfected the mutants to HEK293 cells and carried out co-immunoprecipitations with transportin1 and with importin β1. Surprisingly, an interaction was also observed with importin β1, indicating that necdin can utilize multiple import pathways to the nucleus (Figure 4F). The D234P mutant revealed an increased interaction with both the karyopherins (Figure 4F). The G233A M9-consensus disrupting mutation retained a capacity to interact with transportin1, further confirming that the binding site is not a classical M9-like sequence.

### 4. Necdin localization affects cell viability

**Figure 5:**
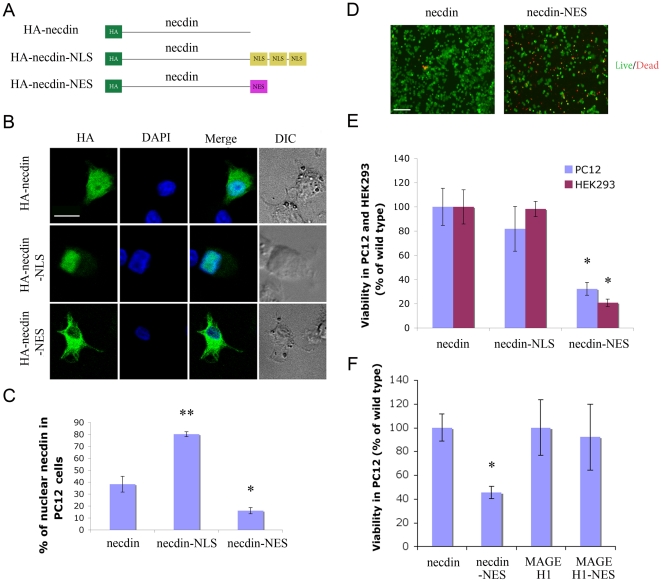
Exclusion of necdin from the nucleus causes cell death. **A**. Schematic of necdin constructs, fused at the C-terminus to either three repeats encoding DPKKKRKV (NLS) or YLVQIFQELTL (NES). **B**. Representative confocal images of PC12 cells 48 hr after transfection, fixed and stained as indicated. Scale bar 10 µm. **C**. Quantification of nuclear localization of the indicated constructs in PC12 cells, * denotes p<0.05, ** denotes p<0.01, n = 16. **D**. Live/dead staining of PC12 transfected as indicated and plated on Poly-L lysine coated cover slips. 30 hours after transfection, cells were washed and incubated with 1 µM Calcein AM and 1 µM EthD-1 in D-PBS for 25 minutes, followed by fluorescence imaging. Live cells are green, dead cells are red. Scale bar 100 µm. **E**. Cell death in necdin-NES transfected PC12 and HEK293 cells as observed by XTT assay. **F**. Viability of PC12 cells transfected with the indicated constructs, 48 hours after transfection. * indicates p<0.02, n = 3.

We further attempted to control subcellular localization of necdin by fusing the ORF with either NLS or NES sequences (Figure 5A). Figure 5B shows representative images of necdin localization in PC12 cells 48 hours after transfection with each of these constructs. Quantification of the ratio of nuclear versus cytoplasmic necdin showed that the NLS and NES sequences were effective in increasing or decreasing necdin nuclear localization, respectively (Figure 5C). Surprisingly, necdin-NES transfected cultures exhibited significantly elevated levels of cell death (Figure 5D–F). To verify that this cell death is caused specifically by necdin, rather than being a non-specific effect due to overexpression of an NES-linked protein, we generated NES fusion constructs of MAGE H1, a necdin homolog that also interacts with the p75 receptor [17]. MAGE H1-NES did not affect cell viability in transfected PC12 cells (Figures 5F and S2), confirming the specificity of the cell death effect observed upon necdin exclusion from the nucleus.

## Discussion

We carried out a large-scale analysis of necdin interactions based on an RRS protein-protein interaction screen in yeast. The network of necdin interactors thus identified displays a high degree of modularity and decomposes into nine main connectivity modules, with defined functional roles and cellular localizations. The RRS method does not require that the fusion proteins be targeted to the nucleus, potentially revealing a wider range of interactions than conventional two-hybrid methods [27]. However, we did not find all the previously published necdin interactors in our screen, most likely since screen coverage and library complexity were far from saturation, and certain interactions might not be compatible with RRS. As in other high-throughput protein interaction screens, some of the putative targets may be false positives or indirect binding partners, necessitating validation with other methods. Network analyses can provide a useful first step in such a validation [28], [29], especially when focused on functional modules of restricted size [30]. We therefore mapped all putative interactions detected in the screen onto a combined network constructed from previously published interactions and examined the connections to structurally coherent modules. The detection of several putative connections to a given module raises the confidence of the detected interaction, and an enriched GO annotation for a necdin-linked module increases the level of confidence yet further. Such interactions are expected to be good candidates for further experimental validation.

The discovered modules confirm necdin's diverse roles as both a cytoplasmatic adaptor (the Mage, Grin-Ywhab, P75, Transportin and Htt modules) and a nuclear factor (the p53-Crebbp module). Connections between the modules can shed futher light on possible roles of necdin, for example the connection between sirtuin1 (Sirt1) and p53. Necdin has been shown to regulate the acetylation status of p53 via Sirt1 to suppress p53-dependent apoptosis in postmitotic neurons [31], a pathway spanning both the p53-Crebbp and the clock modules. Another novel role for necdin is suggested by the newly discovered interactions with a huntingtin module. Necdin plays a role in intracellular p75 signaling endosomes [32], [33], and the RRS and network results suggest that it may be of interest to seek for a similar role in huntingtin complex mediated vesicular trafficking [34], [35].

Different studies have revealed changes in the subcellular localization of necdin upon signaling events or interactions with both nuclear and cytoplasmic proteins [11], [15], [22], [36]. Our findings indicate that interactions with transportins 1 and 2 can play a role in necdin transport between nuclear and cytoplasmic compartments. Moreover, the interaction of different necdin mutants with importin β1 suggests that necdin can use multiple routes of nucleocytoplasmic transport. Forced exclusion of necdin from the nucleus by fusion with an NES sequence caused significant death in transfected cells, further highlighting the importance of controlling subcellular localization of necdin. It is intriguing to speculate that the necdin-NES induced cell death might be due to nuclear exclusion of necdin-interacting survival regulating proteins such as p53 or CCM2 [31], [37], [38]. Since one of necdin's suggested roles is as an anti-apoptotic factor [8], [39], [40], and necdin expression is regulated by transcription factors implicated in cell survival [41], [42], the mechanisms by which changes in necdin subcellular localization affect viability will be an interesting direction for future work.

## Materials and Methods

### RRS screen, bait and cDNA cloning and sequence analysis

Protein-protein interaction screens were conducted using the yeast Ras Recruitment System (RRS) [18]. Briefly, full-length mouse necdin was cloned into the BamHI site of pADH, creating a fusion at the C-terminus of RAS lacking a carboxy-terminal CAAX. Plasmids encoding this necdin bait or mGAP were co-transformed into *cdc25-2* yeast cells for screening of a mouse embryonic head cDNA library in pMyr as previously described [17].

### Interaction network assembly and detection of modules

Mouse protein-protein interactions for necdin were extracted from the IntAct database [19]. Additional published mammalian interactors were added from the literature, as well as new interactors identified in our screen. All available interactions were combined to create the overall network. Modules, defined as sub-networks with a high density of within-group edges and a lower density of between-group edges, were identified using the betweenness centrality clustering algorithm [43] as implemented in GraphWeb [44]. The algorithm successively removes edges with the largest betweenness centrality measure, edges that are expected to connect densely connected sub-networks, until 75% of the network edges are left. Modules are the resulting connected components. Network modularity was quantified by the Q modularity index [21], calculated for optimal module partition versus values for randomized network versions generated with mfinder [45], and containing the same number of nodes, edges and edges per node. GO annotations for modules were obtained using g:Profiler [46], and VisAnt [47] was used for network visualization.

### Expression vectors and mammalian cell expression

All cloned genes were from mouse origin, unless otherwise noted. We generated fusion constructs of the necdin ORF followed by either a triplicate SV40 NLS (sequence encoding DPKKKRKV repeated three times) in pHcRed1-nuc (Clontech), or a Keap1 NES (sequence encoding YLVQIFQELTL) [48]. HA-necdin was cloned into NheI-XhoI sites in pHcRed1-nuc after excision of the RFP-encoding sequence, such that the triplicate NLS sequence in the plasmid was in the same reading frame. For NES cloning, HA-necdin-NES was cloned into pHcRed1-nuc after excision of the RFP-encoding sequence, with a stop codon in frame after the NES sequence. For wild type necdin, HA-necdin was cloned into pHcRed1-nuc after excision of the RFP-encoding sequence, with a stop codon in frame after the necdin ORF. Human MAGE H1 and human MAGE H1-NES were cloned in a similar way. The original pHcRed1-nuc was used as a control vector. For necdin M9 mutations, HA-necdin was cloned into BamHI-XhoI sites in pcDNA3 (Invitrogen) and used as a template for site-directed mutagenesis. Primers with point mutations were used for PCR with Pfu polymerase (Promega). DNA was digested with DpnI (Fermentas), subcloned and sequenced to verify the mutations. PC12 and HEK293 cells were cultured and transfected as previously described [17], [33].

### Antibodies, immunoprecipitation (i.p.), Western blots, and immunofluorescence

I.p.'s, Western blots and immunofluorescence imaging were performed as previously described [17], [33]. The following antibodies were used: for i.p. anti-HA (Covance) at 3 µg per sample; for Western blots, anti-HA monoclonal (Covance) at 1∶1000 dilution; anti-Transportin1 (MBL) at 1∶1000, anti-Importin β1 (Affinity Bioreagents) at 1∶1000, and HRP-conjugated secondary antibodies from Bio-Rad at 1∶10,000. Antibodies for immunofluorescence were anti HA (Covance) at 1∶1000, and fluorophor-conjugated secondaries (Jackson ImmunoResearch) at 1∶1000, or 1∶500 for cy5.

### Imaging and quantification

We used an Olympus laser-scanning confocal microscope (V500 equipped with an Olympus IX70 inverted microscope, objective ×40) to image DAPI for nuclear staining and anti-HA for necdin staining. Quantification of necdin nuclear localization was carried out after manual segmentation, marking the cellular outline and the nuclear outline of all cells without an abnormal shape within each field. Abnormal shapes included cells with no nuclear staining and cells without an apparent cytoplasm, as well as cells that were not in focus in the chosen z-section. A Matlab script was used to quantify the total intensity within the cellular outline and the nuclear outline and then to calculate the ratio of total intensity within the nucleus to the total intensity within the entire cell, representing the fraction of the protein in the nucleus. Background intensity was subtracted from all values.

### Cell viability assays

For XTT assay (Biological Industries), cells were supplemented with 2× medium and 1× tetrazolium salt reaction mixture (XTT reagent) with 1∶50 PMS (activation reagent). Cells were incubated at 37°C for 1–2 hours, followed by absorbance measurement at 450 nm, with subtraction of background measurements carried out at 630 nm. Live/dead assay (Molecular Probes) was performed according to the manufacturer's protocol.

### Statistics

All parametric data are presented as mean ± standard errors (SE). Differences between mean values from each experimental group were examined with Students t test and were considered significant if p<0.05.

## Supporting Information

Figure S1 **Mouse protein interaction network.** The network was parsed from the IntAct database, complemented with the interactions detected in the present screen, and contains 2687 proteins (nodes) with 3817 interactions (edges). Necdin (NDN) is displayed in red. Blue edges denote published interactions, red edges are interactions detected in the present RRS screen.
(TIF)

Figure S2 **MAGE H1 and MAGE H1-NES localization in PC12 cells.** PC12 cells were transfected with MAGE H1 or MAGE H1-NES, and fixed after 48 hours. Cells were immunostained with anti HA (red) and DAPI (blue), and viewed under a confocal microscope.
(TIF)

Table S1 **Results of Necdin RRS Screen.**
(XLS)

Table S2 **The necdin network.**
(XLS)

Table S3 **Module-enriched GO annotations.**
(DOC)
