# Supplementary figures and images for: Functional Consequences of Necdin Nucleocytoplasmic Localization
Source: PLoS One. 2012 Mar 19;7(3):e33786. doi: 10.1371/journal.pone.0033786 (PMC3307762; doi:10.1371/journal.pone.0033786)

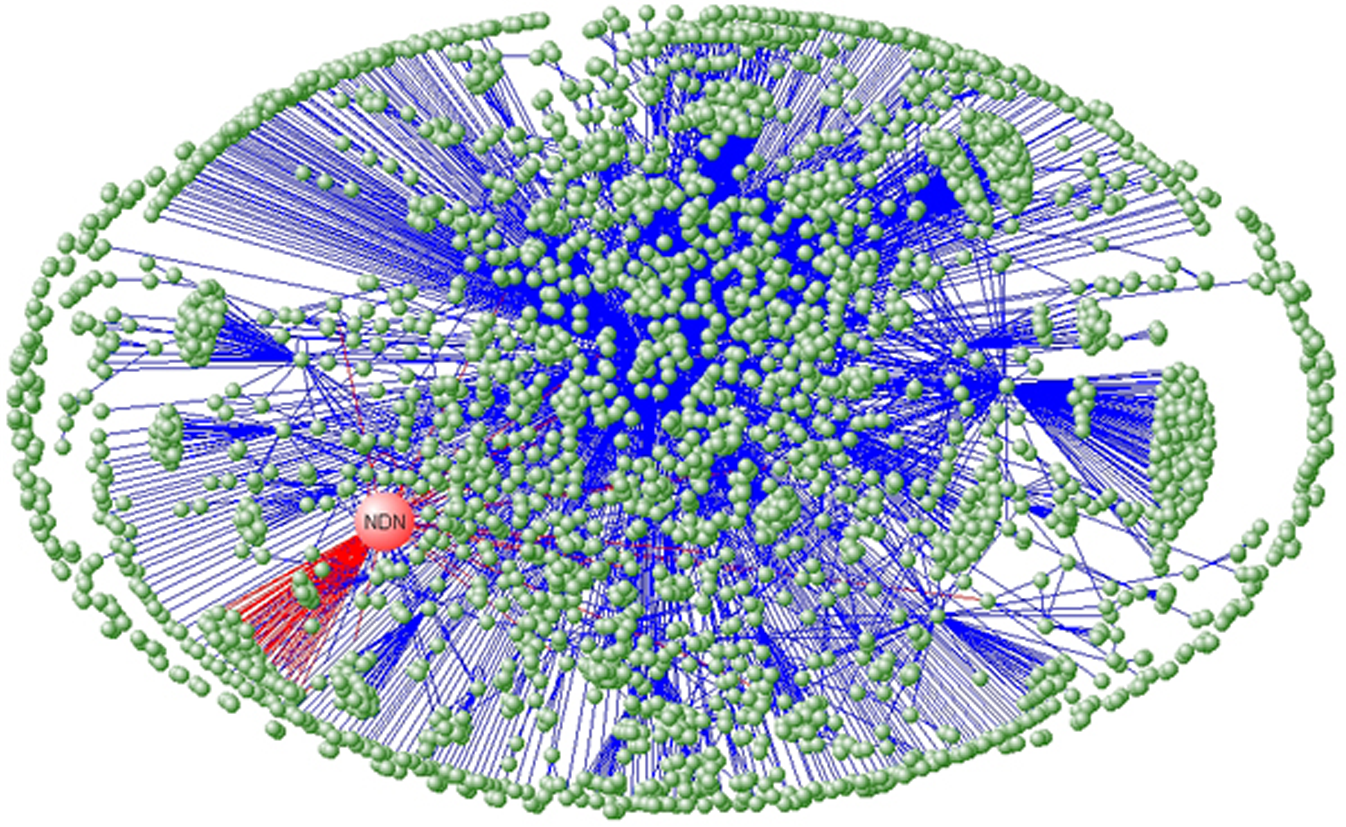

Supplement: Figure S1 — Mouse protein interaction network. The network was parsed from the IntAct database, complemented with the interactions detected in the present screen, and contains 2687 proteins (nodes) with 3817 interactions (edges). Necdin (NDN) is displayed in red. Blue edges denote published interactions, red edges are interactions detected in the present RRS screen. (TIF) [file pone.0033786.s001.tif]

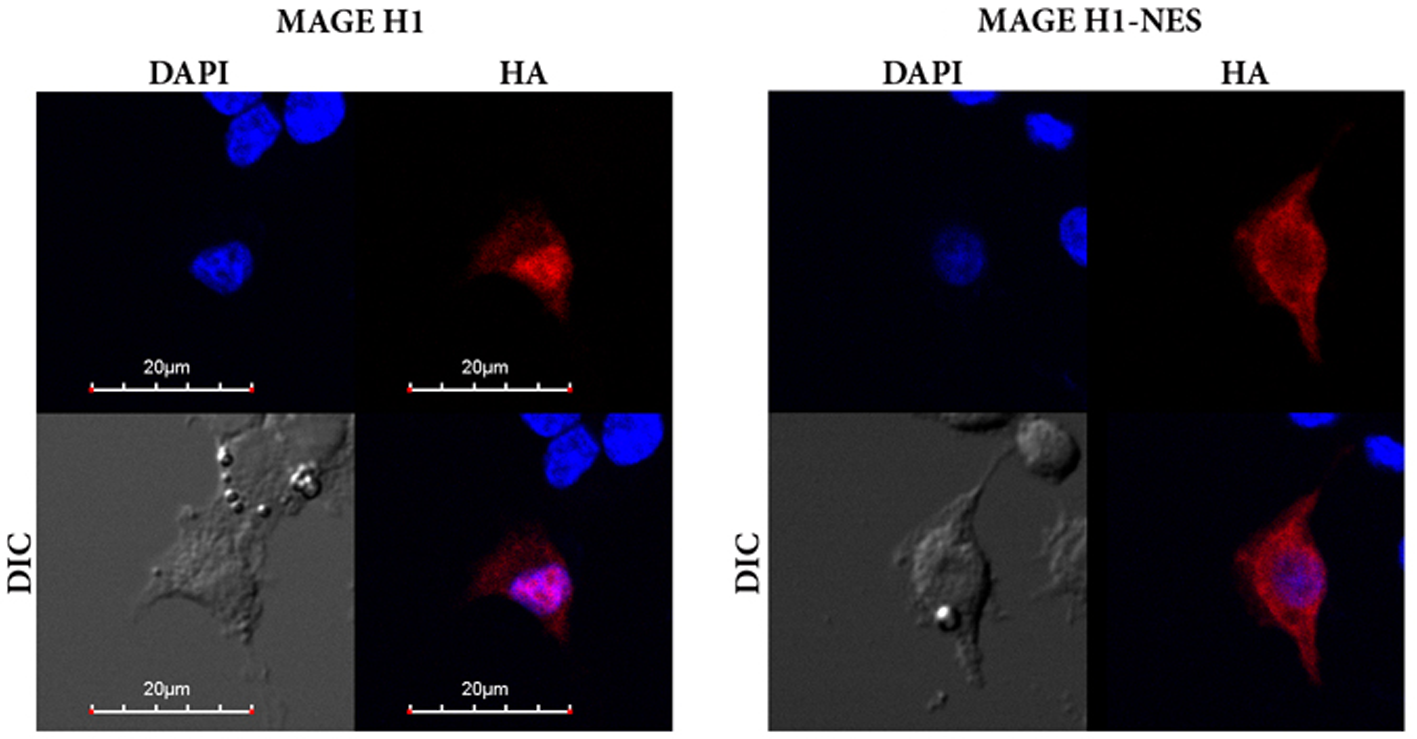

Supplement: Figure S2 — MAGE H1 and MAGE H1-NES localization in PC12 cells. PC12 cells were transfected with MAGE H1 or MAGE H1-NES, and fixed after 48 hours. Cells were immunostained with anti HA (red) and DAPI (blue), and viewed under a confocal microscope. (TIF) [file pone.0033786.s002.tif]
