# Supplementary material for: Functional Consequences of Necdin Nucleocytoplasmic Localization
Source: PLoS One. 2012 Mar 19;7(3):e33786. doi: 10.1371/journal.pone.0033786 (PMC3307762; doi:10.1371/journal.pone.0033786)
Supplement: Table S3 — Module-enriched GO annotations. (DOC) [file pone.0033786.s005.doc]

**Table S3: Module-enriched GO annotations**

| **Module** | **Enriched GO annotations *** |
| --- | --- |
| **necdin** | none |
| **P75** | Cell communication **(**biological process GO:0007154  p=9.17e-7) |
| **Grin-Ywhab** | Response to chemical stimulus (biological process GO:0042221, p=8.35e-9) |
| **p53-CREBBP** | Establishment and maintenance of chromatin architecture (biological process GO:0006325, p=3.19e-7) and nucleus (cellular component GO:0005634, p=1.68e-10) |
| **Transportin** | RNA binding (molecular function GO:0003723, p=1.95e-10**)** |
| **Huntingtin** | Axon cargo transport (biological process GO:0008088 p=4.42e-6) |
| **MAGE D1** | Embryonic limb morphogenesis (biological process GO:0030326, p=1.15e-5). |
| **APP** | Central nervous system development (biological process GO:0007417, p=3.67e-8) |
| **Clock** | Rhythmic process (biological process GO:0048511, p=2.78 e-8) and transcription factor binding (molecular function GO:0008134, p=3.96e-10) |

*** These modules are enriched in several GO annotations; the table shows the representative ones.**
